# Metabolic and functional specialisations of the pancreatic beta cell: gene disallowance, mitochondrial metabolism and intercellular connectivity

**DOI:** 10.1007/s00125-020-05205-5

**Published:** 2020-09-07

**Authors:** Guy A. Rutter, Eleni Georgiadou, Aida Martinez-Sanchez, Timothy J. Pullen

**Affiliations:** 1 Section of Cell Biology and Functional Genomics, Department of Metabolism, Digestion and Reproduction, Imperial College London, Hammersmith Hospital, Du Cane Road, London, W12 0NN UK; 2 Lee Kong Chian School of Medicine, Nanyang Technological University, Singapore, Republic of Singapore; 3 Department of Diabetes, School of Life Course Science, Faculty of Life Science and Medicine, King’s College London, London, UK

**Keywords:** Beta cells, Disallowed genes, Insulin secretion, Interconnectivity, Mitochondria, Review, Type 2 diabetes

## Abstract

All forms of diabetes mellitus involve the loss or dysfunction of pancreatic beta cells, with the former predominating in type 1 diabetes and the latter in type 2 diabetes. Deeper understanding of the coupling mechanisms that link glucose metabolism in these cells to the control of insulin secretion is therefore likely to be essential to develop new therapies. Beta cells display a remarkable metabolic specialisation, expressing high levels of metabolic sensing enzymes, including the glucose transporter GLUT2 (encoded by *SLC2A2*) and glucokinase (encoded by *GCK*). Genetic evidence flowing from both monogenic forms of diabetes and genome-wide association studies for the more common type 2 diabetes, supports the importance for normal glucose-stimulated insulin secretion of metabolic signalling via altered ATP generation, while also highlighting unsuspected roles for Zn^*2+*^ storage, intracellular lipid transfer and other processes. Intriguingly, genes involved in non-oxidative metabolic fates of the sugar, such as those for lactate dehydrogenase (*LDHA*) and monocarboxylate transporter-1 ([MCT-1] *SLC16A1*), as well as the acyl-CoA thioesterase (*ACOT7*) and others, are selectively repressed (‘disallowed’) in beta cells. Furthermore, mutations in genes critical for mitochondrial oxidative metabolism, such as *TRL-CAG1–7* encoding tRNALeu, are linked to maternally inherited forms of diabetes. Correspondingly, impaired Ca^2+^ uptake into mitochondria, or collapse of a normally interconnected mitochondrial network, are associated with defective insulin secretion. Here, we suggest that altered mitochondrial metabolism may also impair beta cell–beta cell communication. Thus, we argue that defective oxidative glucose metabolism is central to beta cell failure in diabetes, acting both at the level of single beta cells and potentially across the whole islet to impair insulin secretion.

**Electronic supplementary material:**

The online version of this article (10.1007/s00125-020-05205-5) contains a slideset of the figures for download, which is available to authorised users.

**Figure:**
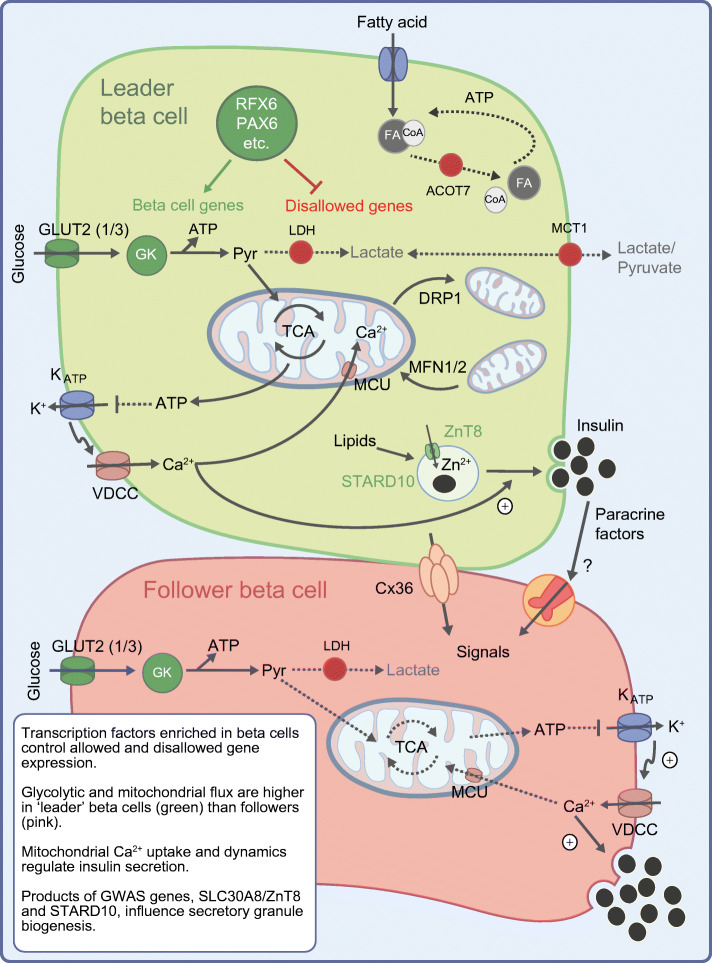
Graphical abstract

## Specialisations of beta cell metabolism

The beta cell is a glucose sensor par excellence, allowing small fluctuations in circulating levels of the sugar to be tuned to insulin output. Certain amino acids, including those that enhance mitochondrial metabolism (e.g. glutamine and leucine) [1], also stimulate insulin release, a response that may be particularly important during fetal development [2]. Fatty acids also stimulate insulin secretion under some circumstances, but can be inhibitory [3].

**Fig. 1:**
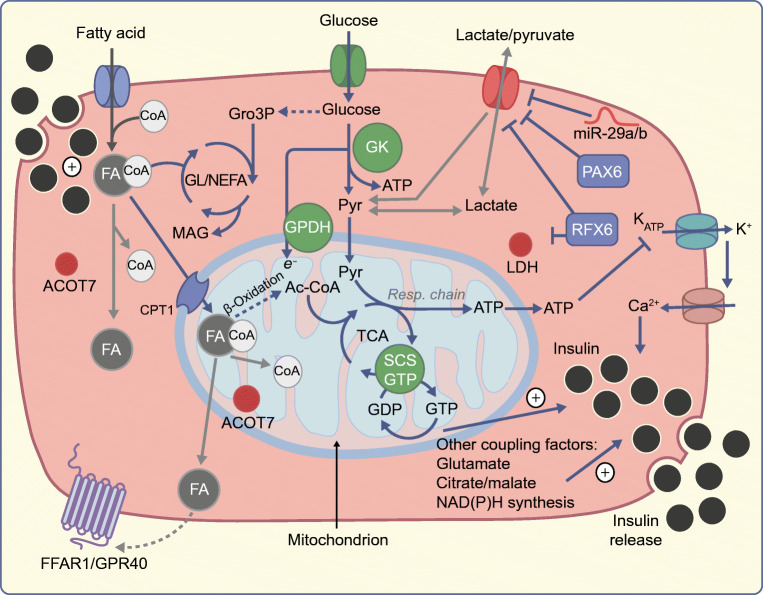
Signalling mechanisms and the role of disallowed genes in beta cell insulin secretion in response to glucose (GSIS). See the main text for further details. GTP is proposed to stimulate insulin release in the cytosol. Products of disallowed genes involved in insulin secretion are represented in red. Lack of lactate dehydrogenase (LDH) and monocarboxylate transporter-1 (MCT-1/SLC16A1) prevents the conversion and extracellular entry, respectively, of lactate and pyruvate which would otherwise prompt inappropriate insulin release. NEFA are activated to FA-CoA in the cytoplasm and can access the mitochondria through carnitine palmitoyltransferase I (CPT-1), where β-oxidation generates Ac-CoA that incorporates into the TCA cycle to potentially enhance insulin secretion. In the cytosol, a glycerolipid/NEFA cycle (GL/NEFA), fatty acids (FA) are esterified with glucose-derived glycerol-3-phosphate (Gro3P) to generate monoacylglycerol (MAG), which enhances insulin release. NEFA could potentially (grey dotted arrow) be released from the beta cell and agonise free fatty acid receptor 1 (FFAR1/GPR40). Low ACOT7 limits the FA-CoA hydrolysis that would result in a lower FA-CoA/NEFA ratio in the cytoplasm or mitochondria. This could affect β-oxidation, the GL/NEFA cycle and the activation of FFAR1 and thus prevent undesired secretory granule release. Examples of transcription factors contributing to gene disallowance are depicted in blue (RFX6, PAX6) and miRNAs are shown in red (miR-29a/b). Ac-CoA, Acyl-CoA; GK, Glucokinase; Pyr, pyruvate; SCS-GTP, succinyl-CoA synthetase; TCA, tricarboxylate cycle. This figure is available as part of a downloadable slideset

Beta cell metabolism of glucose is central to secretion, and these cells express critical ‘glucose sensors’, including the glucose transporter GLUT2 (*Slc2a2*) in rodents (GLUT1 [*SLC2A1*] and GLUT3 [*SLC2A3*] are also expressed in human beta cells) [4]. More crucially for flux control, the low affinity/high *K*_M_ (Michaelis–Menten constant) glucose phosphorylating enzyme, glucokinase (*Gck*), ensures that circulating glucose concentrations are matched to metabolism, which, via changes in electrical activity mediated by ATP-sensitive K^+^ (K_ATP_) channels and Ca^2+^ influx, leads to insulin secretion [5]. Thus, a ‘triggering’ pathway for secretion, largely driven by glucose-induced increases in the intracellular ATP/ADP ratio, plays a cardinal role in glucose-stimulated insulin secretion (GSIS). Additional ‘amplifying’ pathways ensure that glucose also enhances secretion independently of the above pathway [6]. These are less well understood, but enhanced production of mitochondrial metabolites, including glutamate, citrate and reducing equivalents (generated as a result of the activation of metabolic cycles dependent upon mitochondria), notably, NAD(P)H, are all implicated. Work by Kibbey and colleagues [7], also suggests that activated mitochondrial GTP synthesis is a part of this mechanism (Fig. 1).

The existence of variants in genes associated with monogenic forms of diabetes (neonatal diabetes or MODY) [8] provides ample evidence for the importance of several of the key players listed above, including *GCK*, and the K_ATP_ channel subunit genes *KCNJ11* and *ABCC8*. Genome-wide association studies for type 2 diabetes have now also identified ~240 loci and ~400 distinct association signals in the human genome that impact disease risk [9]. Strikingly, the vast majority affect insulin secretion rather than insulin action. Several laboratories, including our own, have provided possible mechanisms of action for some of the implicated genes, including *TCF7L2,* encoding the Wnt-regulated transcription factor [10], *SLC30A8* encoding zinc transporter 8 (ZnT8, the secretory granule zinc transporter) [11], *PAM*, encoding peptidylglycine α-amidating monooxygenase [12] and *STARD10*, encoding an intracellular lipid transporter [13]. The reader is referred to the recent review by Krentz and Gloyn [14] for a more comprehensive survey. A deeper understanding of the roles of these genes, afforded by functional genomics approaches that combine human genetics with interventional (e.g. gene knockout) approaches in tractable systems including mice or CRISPR/Cas9-edited human beta cell lines [14], has provided unexpected insights into beta cell biology, such as the importance of lipid transfer for proinsulin processing [13]. These approaches also offer the exciting prospect of new, and potentially personalised, therapeutic options (‘precision medicine’).

In addition to the roles of genes that are usually highly expressed in beta cells, the relatively weak expression (‘disallowance’) in these cells of several ‘housekeeping’ genes—expressed at high levels in essentially all other cell types in the body, and including founder members of this list of disallowed genes, *Ldha* and *Mct-1* (*Slc16a1*) [15] —is also a defining characteristic of mature beta cells (see below). Inactivation of the latter enzymes is consistent with an unusually high proportion (>85%) of glucose carbon, which is converted to CO_2_ and water via mitochondrial oxidation in these cells [16]. Overexpression of either gene impairs GSIS [17] and unmasks unwanted pathways, including pyruvate-induced secretion [18]. The latter process underlies a genetic trait, exercise-induced hyperinsulism, in carriers of activating variants of the human *SLC16A1* (*MCT-1)* gene [19] (Fig. 1).

A further example of a beta cell ‘disallowed’ gene is *Acot7*, the product of which hydrolyses long-chain acyl-CoAs into NEFA and CoA (Fig. 1). Overexpression of acyl-CoA thioesterase 7 (ACOT7) in beta cell lines, and in primary beta cells in mice in vivo, blunts their insulin secretory response to glucose and fatty acids and results in impaired glucose tolerance [20]. In this case, disallowance appears to reflect ATP sparing for the otherwise futile synthesis and degradation of certain lipid groups [20].

Our laboratory [21] and others [22] have now identified more than 60 beta cell disallowed genes, implicating a range of other cellular processes required for normal insulin secretion and/or the preservation of beta cell mass. The roles and regulation of a subset of these is described in Table 1. The mechanisms involved in the suppression of these genes, and their relevance for beta cell function and failure in diabetes, is currently an area of active research. DNA methylation [23] and histone modifications [24] (Table 1) are well-established mechanisms underlying beta cell-specific gene disallowance. Of note, the transcription factor gene *RFX6*, variants of which were recently identified in man as being responsible for a form of MODY [25], was recently shown to be more weakly expressed in islets from individuals with type 2 diabetes than individuals without the disease [26]. Importantly, inactivation of *Rfx6* in the beta cell in mice both during development and in adult stages leads to impaired function [27]. This reflects impaired expression both of beta cell signature genes and of disallowed genes (below), the regulator regions of which are directly bound by regulatory factor X6 (RFX6). Similarly, another transcription factor important for beta cell development, paired box 6 (PAX6), also plays a pivotal role in maintaining cellular identity and the suppression of disallowed gene expression in adult mice [28, 29]. Like RFX6, PAX6 appears to be able to act ‘bimodally’ to either activate or repress gene expression depending on genomic context.

**Table 1:** Selected islet and beta cell disallowed genes describing putative roles, mechanisms of repression and evidence of increased expression in type 2 diabetes

Gene	Name	Comments	Known mechanisms of repression	Evidence of increase in T2D from LCM [61] or islet [62] data
*Slc16a1*	Monocarboxylate transporter 1	Overexpression causes exercise-induced hyperinsulinism via pyruvate-induced insulin secretion	miRNA-29 family [32, 63] Histone methylation [24]	Islets
*Ldha*	Lactate dehydrogenase A	Combined overexpression with *Slc16a1* causes lactate-sensitive insulin secretion	DNA methylation [23]	LCM
*Acot7*	Acyl-CoA thioesterase 7	Overexpression impairs glucose- & fatty acid-stimulated insulin secretion	Histone methylation [24]	LCM and islets
*Igfbp4*	Insulin-like growth factor binding protein 4	Lower in beta cells than alpha cells [21]. Involved in regulation of proliferation	miRNAs [31]	–
*Mgll*	Monoglyceride lipase	Catalyses hydrolysis of mono-acyl glycerol (MAG), a potential coupling factor in insulin secretion	–	Islets
*Cxcl12*	Chemokine (C-X-C motif) ligand 12	Role in immune regulation, exogenous treatment prevents immune rejection of transplanted islets	–	Islets
*Smoc2*	SPARC related modular calcium binding 2	Role in islets unknown but evidence for mitogenic role in other cells	–	Islets
*Pdgfra*	Platelet derived growth factor receptor, alpha polypeptide	Pro-proliferative role in beta cells, and repression linked to age-related decline in proliferative capacity	miRNAs [63, 31]	LCM
*Hsd11b1*	Hydroxysteroid 11-beta dehydrogenase 1	Greatest downregulation in beta cells vs other tissues [21]. Major regulator of local glucocorticoid signalling	–	–
*IGF1*	Insulin-like growth factor 1	Pro-proliferative role in beta cells	–	Islets
*Yap1*	Yes-associated protein 1	Lower in beta cells than alpha cells [21] Potent driver of proliferation via Hippo pathway	miR-375	–

For other details and further references see [21]. LCM: Laser capture microdissection; T2D, type 2 diabetes

MicroRNAs (miRNAs) are also important contributors to beta cell gene disallowance (Table 1). miRNAs are non-coding RNAs that silence gene expression to fine-tune biological pathways and reinforce cellular identity [30]. Beta cell-specific deletion of DICER, an enzyme essential for miRNA biogenesis, relieved the suppression of several disallowed genes in mice, namely, *Fcgrt*, *Igfbp4*, *Maf*, *Oat*, *Pdgfra* and *Slc16a1* [31]. Whether the more recently identified disallowed genes highlighted in Pullen et al. [21] are also regulated by miRNAs remains to be investigated. Little is known about the identity of the miRNAs targeting these genes in beta cells, though miR-29a/b and miR-34a have been demonstrated to target *Slc16a1* [32], and *Pdgfra* [33], respectively. It is conceivable that a complex network of miRNA-disallowed gene interactions contributes to reinforce beta cell identity by ensuring gene disallowance. Whether other non-coding RNA species (long non-coding RNAs, circular RNAs, etc.) are also involved remains to be explored.

## Mitochondria and insulin secretion

Weak expression in beta cells of *Ldha* and *Mct-1/Slc16a1* emphasises the likely importance of oxidative metabolism of glucose carbons for the normal stimulation of insulin release. Similarly, low expression of *Acot7* underlines the importance of mitochondrial fatty acid metabolism for efficient ATP utilisation. Thus, mitochondrial ATP synthesis in response to elevated glucose or other nutrients is essential to both the triggering and amplifying pathways of insulin exocytosis [34]. There is strong evidence linking the loss or dysfunction of GSIS in beta cells of diabetic models with altered mitochondrial function, where nutrient storage and usage, as well as mitochondrial dynamics and morphology, are affected [35]. A further striking example is provided by hyperglycaemic ‘βV59M’ mice, expressing an activated form of the K_ATP_ channel subunit Kir6.2 [36], where an increase is observed in pyruvate dehydrogenase (PDH) kinase expression (expected to lower PDH activity and hence pyruvate entry into the cycle), as well as lowered levels of several citrate cycle genes.

Several mtDNA (mitochondrial DNA) variations in human populations have been implicated in increased or decreased risk of type 2 diabetes while, in animal models, alterations in beta cell mtDNA led to reduced insulin secretion, hyperglycaemia and beta cell loss [34]. In humans, maternally inherited diabetes and deafness (MIDD) is often linked to an mtDNA A3243G point mutation in the *TRL-CAG1-7* (tRNALeu) gene, responsible for defective mitochondrial metabolism and impaired intracellular Ca^2+^ homeostasis [37].

mtDNA encodes most subunits of the electron transport chain, and inactivation of the mitochondrial transcription factor A (*Tfam*) specifically in mouse beta cells resulted not only in mtDNA depletion and deficient oxidative phosphorylation (OXPHOS) but also in impaired secretion and hyperglycaemia in vivo [38]. Moreover, mutations in the mitochondrial gene encoding frataxin, known for its iron–sulphur cluster activation and respiratory function in mitochondria, are associated with Friedreich’s ataxia (FRDA) [39], which involves mitochondrial iron overload, respiratory chain dysfunction, impaired OXPHOS and ATP production. Importantly, frataxin expression is upregulated by glucagon-like peptide (GLP-1) receptor agonists [40], an effect that may contribute to the glucose-lowering actions of these drugs.

The role of Ca^2+^ accumulation by mitochondria has long been a contested aspect of GSIS. Ca^2+^ uptake into these organelles in living beta cells was initially demonstrated in response to an increase in cytosolic Ca^2+^ through the use of a recombinant mitochondrially-targeted aequorin [41]. Although thought likely to lower mitochondrial membrane potential (Δψ_m_), studies based on the discovery in the 1970s of Ca^2+^-sensitive intra-mitochondrial dehydrogenases in the citrate cycle [42] have suggested a positive role for Ca^2+^ as a stimulator of oxidative metabolism in this compartment. In line with the latter view, deletion of the mitochondrial Ca^2+^ uniporter (MCU) selectively in the beta cell of living mice [43] has revealed that Ca^2+^ uptake is essential for both phases of glucose-stimulated ATP synthesis and insulin secretion in vitro, as well as for the maintenance of normal beta cell mass. However, beta cell-selective *Mcu* null mice showed minor changes in insulin secretion in vivo, suggesting the existence of currently undefined compensatory mechanisms.

**Fig. 2:**
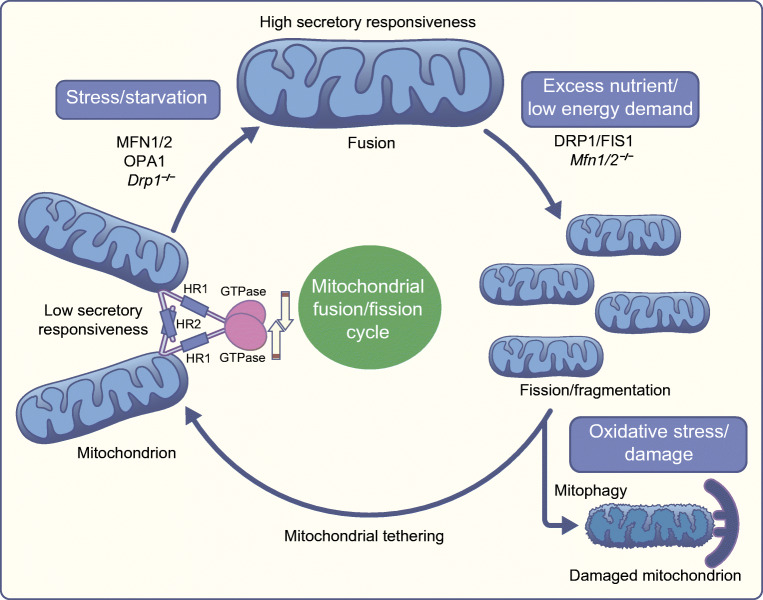
Putative roles for proteins controlling mitochondrial shape and dynamics in beta cells. See the text for further discussion. The outer mitochondrial membrane (OMM) GTPases MFN1 and MFN2 are responsible for the fusion of these membranes on two adjacent mitochondria, while optic atrophy 1 (OPA1), drives inner mitochondrial membrane (IMM) fusion. Heptad repeat domains 2 (HR2) are essential for the initial tethering between adjacent mitochondria, while hydrolysis of the GTPase domain is needed for fusion completion. The latter allows the transfer of mitochondrial membrane components, metabolites and normal mtDNA copies. Elongated mitochondria, with high secretory responsiveness, will undergo fission with the support of DRP1 and FIS1. Fragmentation is an essential process involved in isolating dysfunctional mitochondrial units or mutant mtDNA copies from the mitochondrial network. During mitochondrial division, organelles moderately malfunctioning or damaged (depolarised) due to oxidative stress will undergo autophagy, a process also referred to as mitophagy. Functional mitochondria will instead either remain fragmented (low secretory responsiveness) during high nutrient supply conditions or will fuse with neighbouring organelles when the cell is under high energy demand (starvation). Studies showed that deletion or silencing of *Drp1* (*Drp1*^*−*^*/*^*−*^) or *Mfn1* and *Mfn2 (Mfn1/2*^*−*^*/*^*−*^*),* affect insulin secretion and glucose homeostasis in mice. This figure is available as part of a downloadable slideset

Beta cell mitochondria often exist as densely interconnected tubules that continually undergo interconversions with more granular forms via fission and fusion cycles that are under the control of specific regulatory proteins (Fig. 2). In most cell types, this dynamic process is influenced by nutrient supply as well as extra- or intracellular factors that are critical to cell survival. This is likely also to be the case in beta cells [44] and may be of particular relevance given the specialised roles of nutrient metabolism in these cells. Given that there is likely to be a close association between mitochondrial morphology and function, altered mitochondrial dynamics may well contribute to defective insulin secretion in diabetes. Indeed, several studies have demonstrated that mitochondrial morphology and function are altered in beta cells in diabetic animal models (e.g. the Zucker Diabetic Fatty rat) [42] and beta cell-derived lines [34].

Beta cells from patients with type 2 diabetes also display a marked change in mitochondrial function and morphology, including fragmentation and disruption of cristae morphology [45]. These changes are associated with reduced insulin secretion, a lower ATP/ADP ratio and impaired polarisation of the mitochondrial inner membrane (i.e. the generation of a Δψ_m_ to drive electron transport chain activity) [45]. However, mitochondrial volume density in beta cells from individuals with type 2 diabetes was significantly increased in comparison with healthy or type 1 diabetic donors [46].

Recent results from Ku and colleagues [47], and ourselves [48] demonstrate that the balance between mitochondrial fission and fusion (and hence the maintenance of an appropriately interlinked mitochondrial network) is critical for normal beta cell fuel sensing. Thus, deletion or silencing of one or more of these factors (e.g. *Drp1*, also known as *Dnm1l*) which controls mitochondrial fission) [47, 49] or the mitofusins *Mfn1* and *Mfn2* (which control fusion) [48] both exert profound effects on beta cell mass, insulin secretion and glucose homeostasis in mice. Similarly, deletion of the dynamin-related GTPase optic atrophy protein 1 (OPA1), responsible for fusion of the inner mitochondrial membrane, from beta cells, results in respiratory chain defects and impaired insulin secretion [50]. Of note, human syndromes such as multiple symmetrical lipomatosis (Madelung’s disease), caused by mutations in *MFN2,* appear chiefly to lower insulin sensitivity [51]. Therefore, ablation of both mitofusins may have a greater deleterious impact on beta cell function and survival rather than targeting and inactivating a single mitofusin gene. Interestingly, in studies from Shirihai and colleagues [52], promotion of a fragmented phenotype in cardiomyocyte-derived C2C12 cells resulted in a marked reduction in mitochondrial Ca^2+^ accumulation, hinting that similar changes may impair the uptake of these ions into mitochondria in beta cells, with consequences for glucose metabolism and insulin secretion. Nonetheless, the role and regulation of mitochondrial fission and fusion factors in the beta cell in diabetes mellitus remain to be fully elucidated.

## A role for mitochondria in beta cell heterogeneity and intercellular connectivity?

**Fig. 3:**
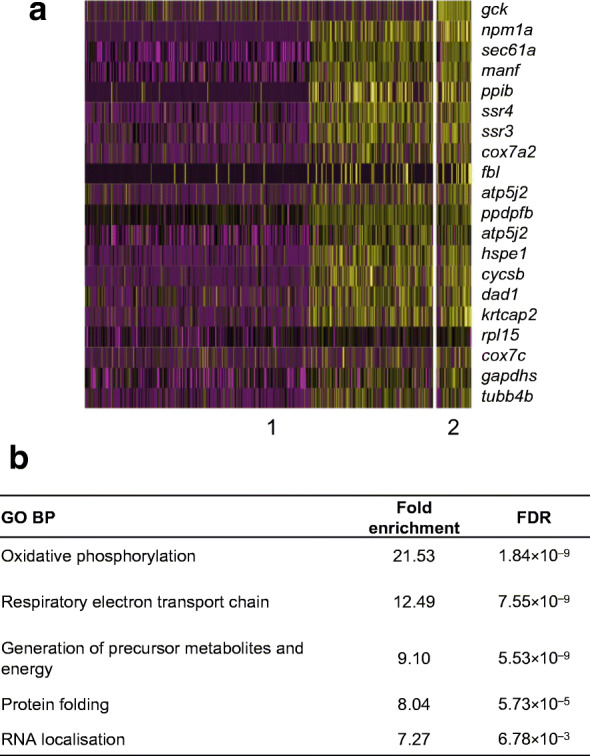
Single cell RNA-seq analysis of islets from the zebrafish (*Danio rario*) to identify putative hub/leader cells. Cluster analysis was performed based on the co-expression of high *Gck*, but low *Ins1* levels in a subset corresponding to ~10% of all cells. (**a**) Heatmap showing the top 20 genes defining the putative hub cells. Hub and follower cells are defined as ‘1’ and ‘0’, respectively. (**b**) Statistically over-represented Gene Ontology (GO) Biological Process (BP) terms in genes upregulated in putative hub cells. FDR, false discovery rate. Adapted from [59] with permission from Springer Nature, ^©^2019. This figure is available as part of a downloadable slideset

**Fig. 4:**
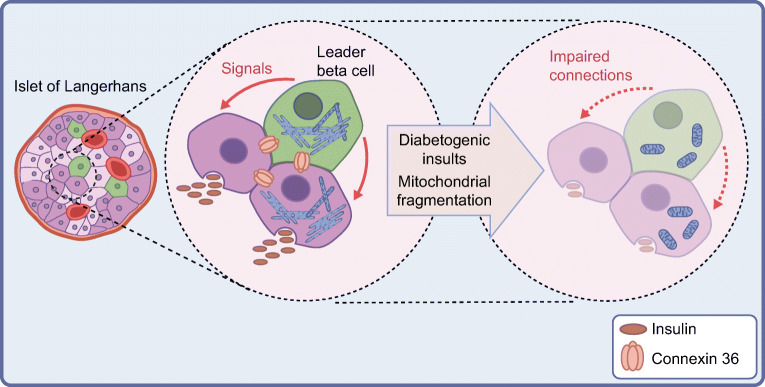
Beta cell network model depicting impairment of insulin secretion following external or genetic alterations to mitochondrial fusion protein expression. Leader (hub) beta cells (green) coordinate pulsatile insulin secretion and signal propagation across an islet through signalling routes such as the gap junction protein connexin 36. Diabetogenic insults or genetic deletion/lowered expression of proteins involved in the mitochondrial fusion process cause the mitochondrial network to rapidly fragment and no fusion occurs while these proteins are absent. This will also lead to progressive reduction in insulin secretion, loss of beta to beta cell interconnection, and development of type 2 diabetes, and may conceivably contribute to secretory insufficiency in type 1 diabetes [60] in some circumstances. For simplicity, non-beta cells are omitted from the islet diagram (left-hand panel). Red structures (no central nucleus) represent capillaries. This figure is available as part of a downloadable slideset

As reviewed by Gutierrez et al. [53], data that first emerged in the 1980s indicated the existence within the islet of multiple beta cell subgroups with distinct metabolic properties. These early results were supported recently by a slew of new studies deploying single cell-omics, notably massive parallel RNA sequencing (RNA-seq) of islet cells from both mice and humans. This validation of the existence of intercellular heterogeneity has raised the possibility that distinct subgroups of beta cells may exert differing roles in the control of islet dynamics (note that the mechanisms though which individual islets are coordinated across the whole pancreas are not addressed here). Supporting this possibility, we have shown that intercellular connectivity is required in the islet for a full insulin secretory response to glucose and incretins [54]. The physical basis of the connections between cells that underlie this property are only partly understood; they include, but are not restricted to, the formation of Connexin 36- (*Cx36*/*Gjd2*) dependent gap junctions [55]. A subset of specialised beta cells, which are unusually highly connected (termed ‘hubs’ or ‘leaders’) [56] and are often the sites of initiation of Ca^2+^ waves, play a disproportionate role in the control of beta cell Ca^2+^ dynamics in the intact islet. Similar findings of functionally distinct (and potentially controlling) beta cell subpopulations have been described by others [57, 58]. Importantly, both glycolytic and mitochondrial metabolism appear to play exaggerated roles in hub/leader cells, as exemplified by RNA-seq analyses in the model zebrafish system (Fig. 3) [59]. Taken together, these data suggest that genetic variants or environmental insults (e.g. gluco/lipotoxicity or inflammation) may act through mitochondrial perturbations to impair beta cell network dynamics and hence insulin secretion (Fig. 4). Enhancing mitochondrial function in this critical subset of cells may thus provide a new therapeutic opportunity in some forms of diabetes.

In summary, defective mitochondrial function is likely to have effects contributing to impaired insulin secretion in type 2 diabetes and, conceivably, in those cases of type 1 diabetes where detectable beta cell mass remains [60]. Importantly, altered mitochondrial function may affect both individual beta cells and the ensemble behaviour that coordinates pulsatile insulin secretion. Although not the subject of the present review, changes in mitochondrial function and structure may also modulate beta cell survival and, hence, mass in both disease settings, for example through the regulation of key pathways such as autophagy, apoptosis and cell senescence. Finally, altered mitochondrial metabolism and signal generation may play important roles in other islet endocrine (and critical non-endocrine) cells to influence the overall pancreatic output of endocrine hormones.

## Electronic supplementary material

Slideset of figures (PPTX 395 kb)

## Abbreviations

Δψ_m_: Mitochondrial membrane potential
ACOT7: Acyl-CoA thioesterase 7
GSIS: Glucose-stimulated insulin secretion
K_ATP_: ATP-sensitive K^+^ (channel)
MCT-1: Monocarboxylate transporter-1
MCU: Mitochondrial Ca^2+^ uniporter
miRNA: MicroRNA
mtDNA: Mitochondrial DNA
OXPHOS: Oxidative phosphorylation
PAX6: Paired box 6
PDH: Pyruvate dehydrogenase
RFX6: Regulatory factor X6
RNA-seq: RNA sequencing
