# Supplementary material for: Metabolic and functional specialisations of the pancreatic beta cell: gene disallowance, mitochondrial metabolism and intercellular connectivity
Source: Diabetologia. 2020 Sep 7;63(10):1990–8. doi: 10.1007/s00125-020-05205-5 (PMC7476987; doi:10.1007/s00125-020-05205-5)
Supplement: Supplementary file 1 — (PPTX 395 kb) [file 125_2020_5205_MOESM1_ESM.pptx]

## Slide 1
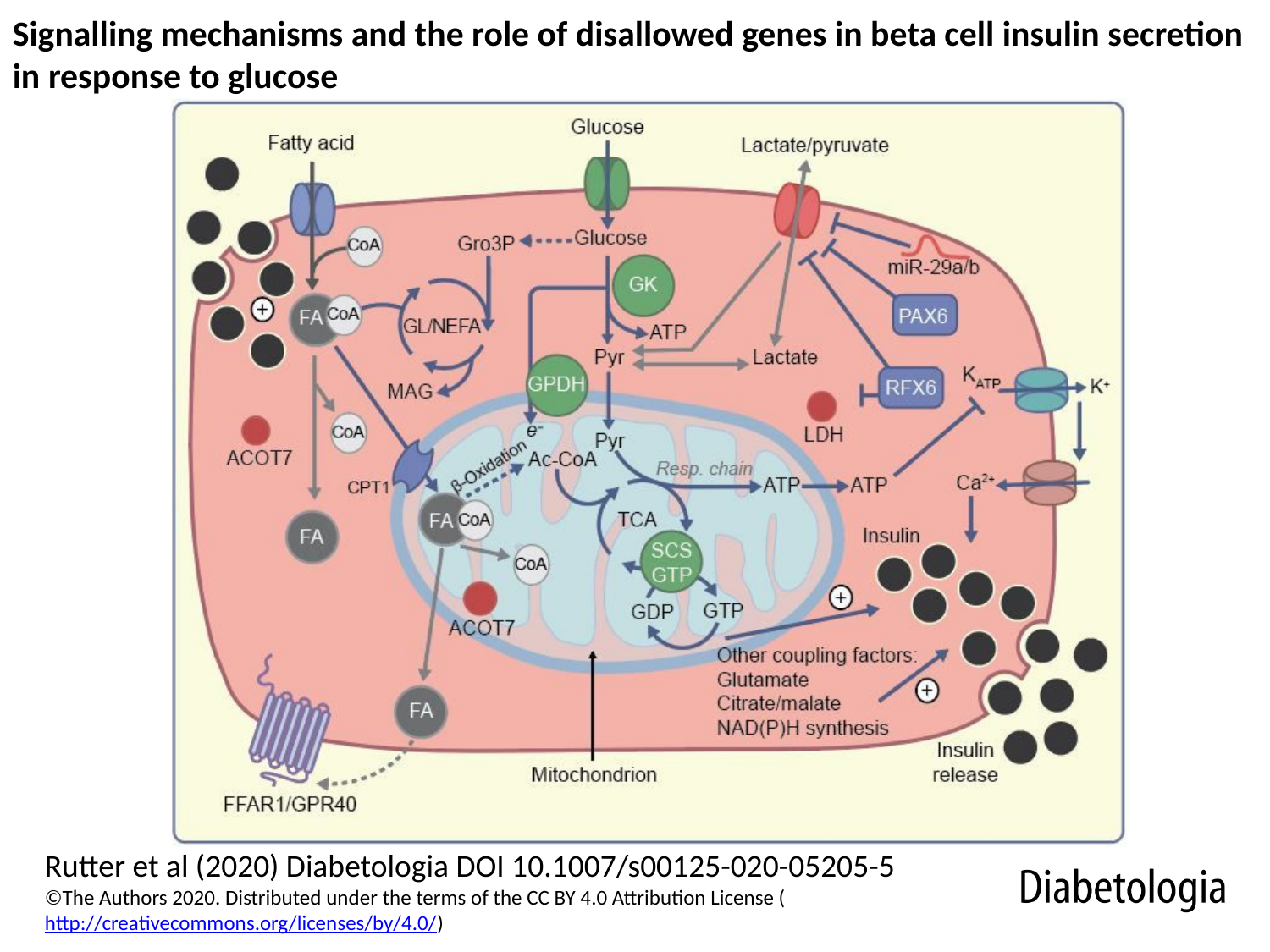

Signalling mechanisms and the role of disallowed genes in beta cell insulin secretion in response to glucose
Rutter et al (2020) Diabetologia DOI 10.1007/s00125-020-05205-5
©The Authors 2020. Distributed under the terms of the CC BY 4.0 Attribution License (http://creativecommons.org/licenses/by/4.0/)

## Slide 2
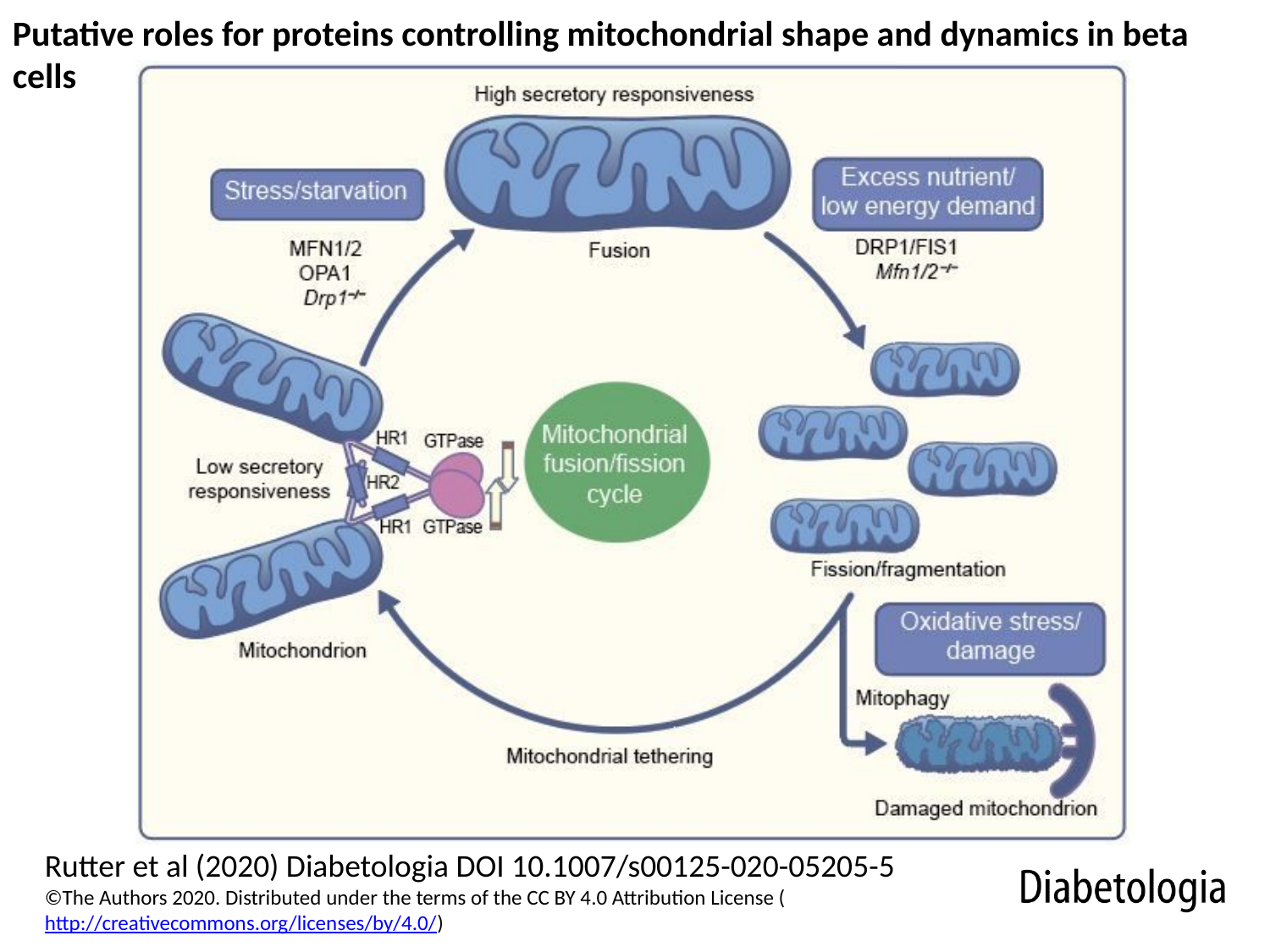

Putative roles for proteins controlling mitochondrial shape and dynamics in beta cells
Rutter et al (2020) Diabetologia DOI 10.1007/s00125-020-05205-5
©The Authors 2020. Distributed under the terms of the CC BY 4.0 Attribution License (http://creativecommons.org/licenses/by/4.0/)

## Slide 3
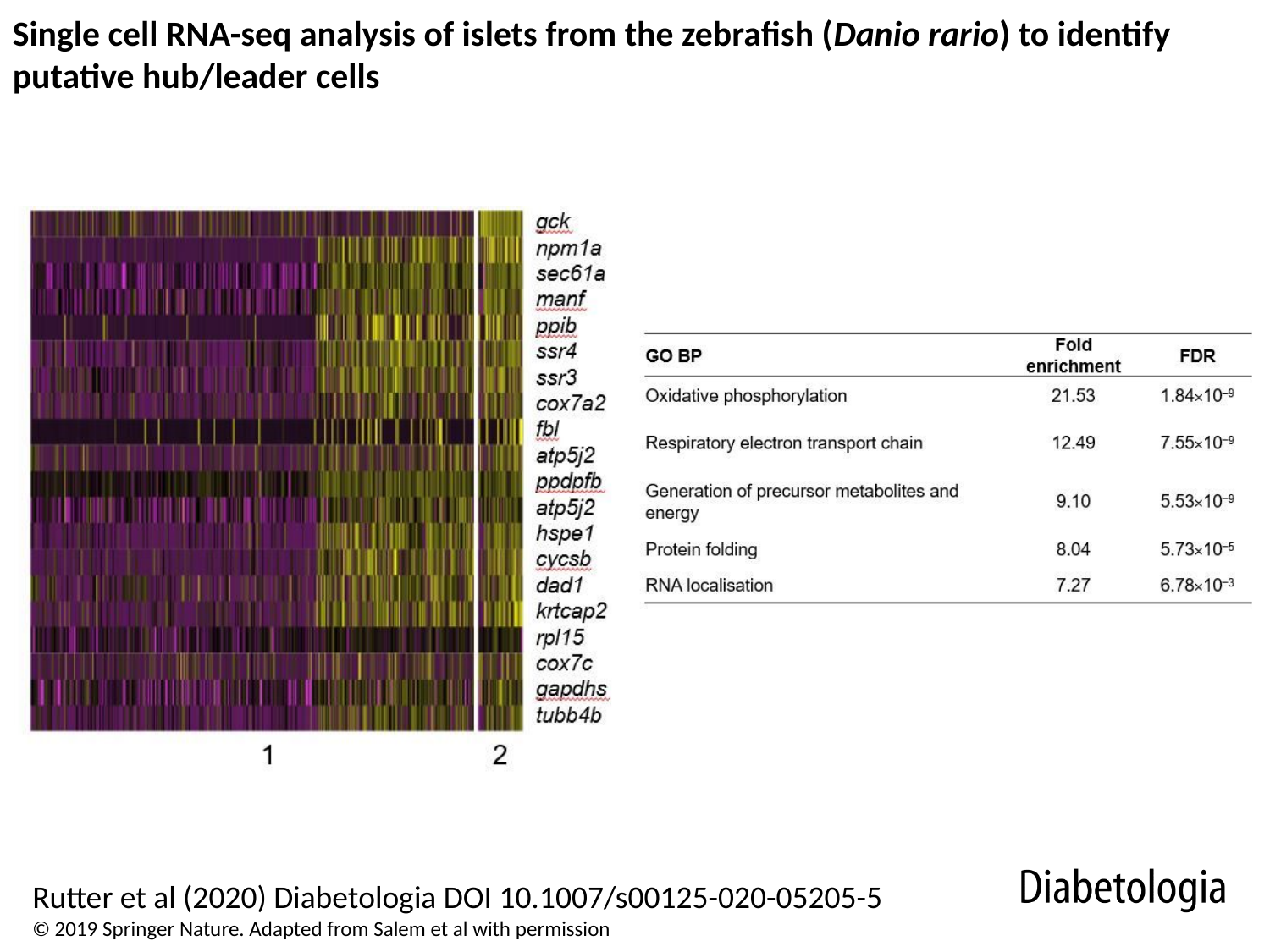

Single cell RNA-seq analysis of islets from the zebrafish (Danio rario) to identify putative hub/leader cells
Rutter et al (2020) Diabetologia DOI 10.1007/s00125-020-05205-5
© 2019 Springer Nature. Adapted from Salem et al with permission

## Slide 4
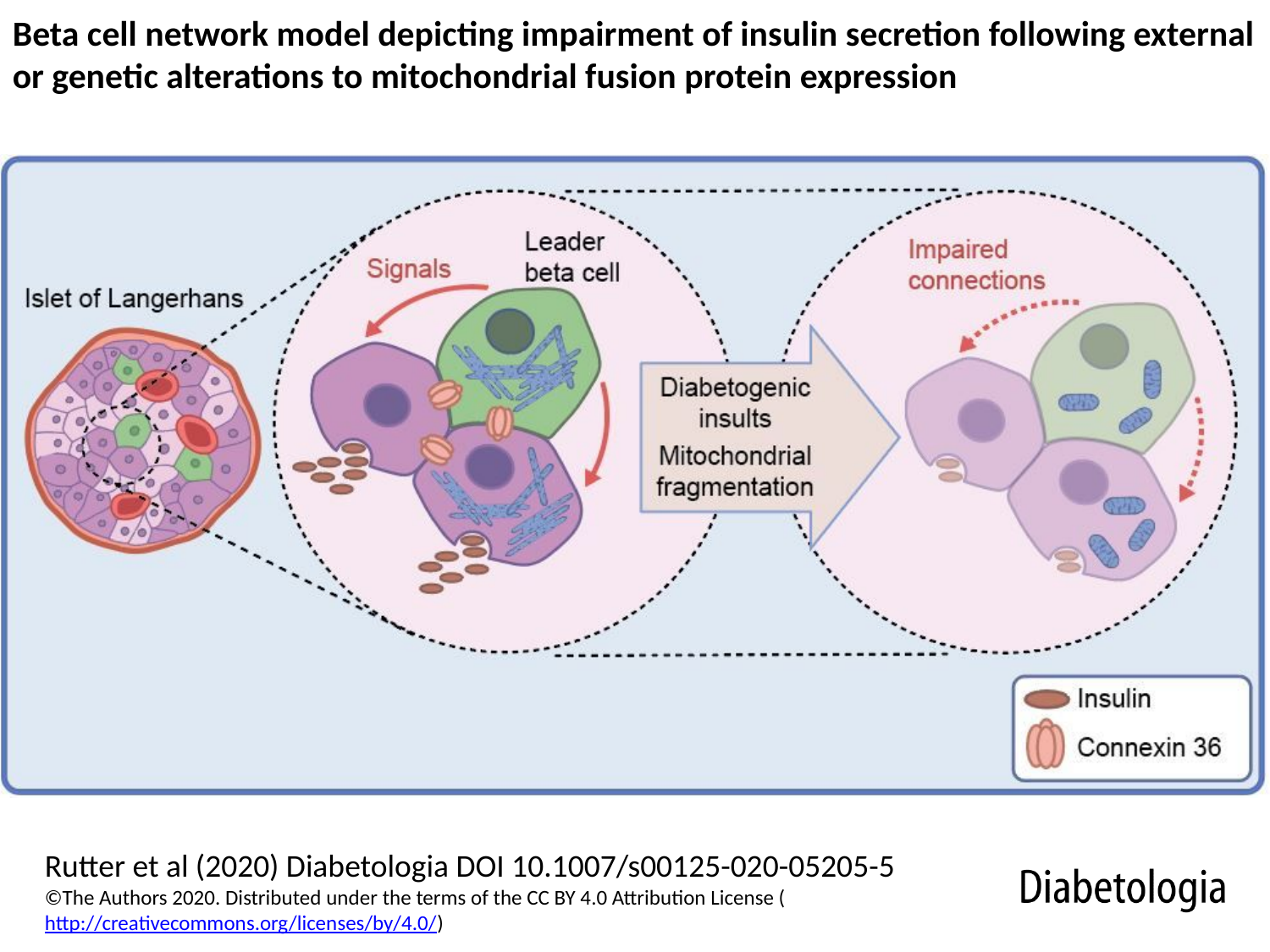

Beta cell network model depicting impairment of insulin secretion following external or genetic alterations to mitochondrial fusion protein expression
Rutter et al (2020) Diabetologia DOI 10.1007/s00125-020-05205-5
©The Authors 2020. Distributed under the terms of the CC BY 4.0 Attribution License (http://creativecommons.org/licenses/by/4.0/)
